# Sick-Room Cookery

**Published:** 1908-09-19

**Authors:** 


### SICK-ROOM COOKERY.

*To the Editor of* The Hospital.

Sir,—We should all be grateful to Dr. Saundby for laying some stress on the importance of a knowledge of sick-room cookery to the general practitioner, and I, for one, have read his lecture, published in your issue of August 8, with the greatest interest. There are, however, a few points which one would have preferred "expressed differently." Thus I do not think it is at all desirable that a sick person's diet should be more unvarying or monotonous than that of a person in average health. Possibly Dr. Saundby does not mean that, but such at least is the inference one is apt to draw from his sentence. The monotonous milk diet to which many patients are so often condemned (sometimes needlessly) can be varied in a dozen ways with benefit to the patient. Many of us argue that a sick man's taste is for the time being gone, and that it does not matter if his menu is milk, milk, milk eternally all day long. There can be no greater mistake than this, and I have found patients keenly sensible and appreciative of a change in diet. Milk may be flavoured in many ways—as Dr. Saundby suggests by infusing it as tea, or by the addition of some harmless extract, or even, better still, by adding to it one of the old-fashioned "vegetable teas," infusions of vegetable marrow, beans, or tomatoes. A beef-tea made with milk is at once nourishing and palatable, while in most cases where milk is tolerated the clear vegetable soups are equally well borne.

Dr. Saundby does well to point out on what lines the diabetic patient's menu may be varied, but I do not quite agree with him that the cook, in such a case, is forced to leave out the *entremets*. A variety of pleasant dessert dishes may be prepared from almonds and nuts, while blancmanges flavoured with some simple extract and sweetened with levulose may be included in the diabetic's dietary. A very agreeable change is in the shape of wine jellies made with gelatine (or, better still, with agar), while there are many varieties of pleasant-tasting diabetic biscuits on the market. I am not sure, too, whether it is altogether wise to deprive our diabetic patients entirely of sugar. One patient I have in mind has now for several years taken very moderate quantities of cane sugar daily with no marked ill effects. I am not, of course, arguing that we should freely pass the sugar-bowl to our diabetics, but merely that we should consider each patient's individual idiosyncrasy. We know as yet so little about the causation of diabetes that we can hardly generalise on broad lines. A few years ago the candidate who in an examination allowed his diabetics to have potatoes would have been severely criticised to say the least, now it is generally acknowledged that total deprivation of starchy foods is by no means desirable.

One other point you will, perhaps, allow me to touch upon. Dr. Saundby is astonished that some German doctors allow their convalescent patients "scraped ham." In all humility may I ask if he has ever tried it? I have, and I can speak from experience as to its value. Lean ham, scraped till it is very finely divided, is appetising when spread between thin bread and butter, and it is certainly very easily digestible. Some time ago I tested it against a fragment of alleged chicken, taken from the dinner plate of a patient in one of our London hospitals, who was on light diet. The grated ham was digested completely in under six hours, while after twelve hours the test-tube containing the "boiled chicken" still showed fibres of undigested material. Digestion experiments *in vitro*, I quite admit, are misleading, but my experience of grated ham—and also of grated fresh meat, pemmican, and the dried meat which in South Africa is known as "Biltong"—has been very favourable. Sandwiches of grated fresh meat are very useful in anæmic and debilitated conditions, as they are very easily assimilated. The *sine qua non*, of course, is that the meat should be above suspicion. Here, on the continent, where meat is regularly inspected, one can be sure of getting good samples: in England, where even hospital supplies (as I have myself found on several occasions) are contaminated, one has to be very careful in ordering fresh, uncooked meat as a diet for patients.

I have already trespassed too largely on your space, but it appears to me to be desirable that the subject of sick-room cookery should receive more attention than it has hitherto done. You sir, I hold, may do much in the matter, and I would suggest that you include in your valuable sections one dealing with diet and dietetics. It would, I feel sure, be of interest to many practitioners.

I am, etc.,

August 31, 1908. Graduate Student.

THE HOSPITAL September 19, 1908.

